# Long-Term Visual Outcomes for a Treat and Extend Anti-Vascular Endothelial Growth Factor Regimen in Eyes with Neovascular Age-Related Macular Degeneration

**DOI:** 10.3390/jcm4071380

**Published:** 2015-07-08

**Authors:** Sarah Mrejen, Jesse J. Jung, Christine Chen, Samir N. Patel, Roberto Gallego-Pinazo, Nicolas Yannuzzi, Luna Xu, Marcela Marsiglia, Sucharita Boddu, K. Bailey Freund

**Affiliations:** 1 Vitreous, Retina, Macula Consultants of New York, New York, NY 10022, USA; E-Mails: sarahmrejen.uretsky@gmail.com (S.M.); jung.jesse@gmail.com (J.J.J.); marcelamarsiglia@hotmail.com (M.M.); 2 LuEsther T. Mertz Retinal Research Center, Manhattan Eye, Ear, and Throat Hospital, New York, NY 10065, USA; 3 Quinze-Vingts Hospital, DHU ViewMaintain, INSERM-DHOS CIC 1423, 75012 Paris, France; 4 Department of Ophthalmology, New York University School of medicine, New York, NY 10016 USA; E-Mail: sucharita.boddu@med.nyu.edu; 5 Edward S. Harkness Eye Institute Columbia, New York, NY 10032, USA; 6 Department of Surgery, Monash University, Melbourne, VIC 3800, Australia; E-Mail: chris_chen30@hotmail.com; 7 Center for Eye Research Australia, University of Melbourne, East Melbourne, VIC 3002, Australia; 8 Weill Cornell Medical College, New York, NY 10065, USA; E-Mails: snp2002@med.cornell.edu (S.N.P.); nicolas.yannuzzi@gmail.com (N.Y.); 9 Department of Ophthalmology, University and Polytechnic Hospital La Fe, 46026 Valencia, Spain; E-Mail: robertogallegopinazo@gmail.com; 10 The New York Eye and Ear Infirmary, New York, NY 10003, USA; E-Mail: luna.xu.v@gmail.com

**Keywords:** anatomical classification, choroidal neovascularization, fluorescein angiography classification, intravitreal anti-VEGF injections, neovascular age-related macular degeneration, Treat and Extend Regimen

## Abstract

With the advent of anti-vascular endothelial growth factor (VEGF) therapy, clinicians are now focused on various treatment strategies to better control neovascular age-related macular degeneration (NVAMD), a leading cause of irreversible blindness. Herein, we retrospectively reviewed consecutive patients with treatment-naïve NVAMD initially classified based on fluorescein angiography (FA) alone or with an anatomic classification utilizing both FA and optical coherence tomography (OCT) and correlated long-term visual outcomes of these patients treated with an anti-VEGF Treat-and-Extend Regimen (TER) with baseline characteristics including neovascular phenotype. Overall, 185 patients (210 eyes) were followed over an average of 3.5 years (range 1–6.6) with a retention rate of 62.9%, and visual acuity significantly improved with a TER that required a mean number of 8.3 (±1.6) (± standard deviation) intravitreal anti-VEGF injections/year (range 4–13). The number of injections and the anatomic classification were independent predictors of visual acuity at 6 months, 1, 2, 3 and 4 years. Patients with Type 1 neovascularization had better visual outcomes and received more injections than the other neovascular subtypes. There were no serious adverse events. A TER provided sustained long-term visual gains. Eyes with Type 1 neovascularization had better visual outcomes than those with other neovascular subtypes.

## 1. Introduction

Age-related macular degeneration (AMD) is a leading cause of irreversible severe vision loss and blindness in patients 50 and older [1]. Neovascular AMD is responsible for 80% of cases of severe vision loss due to AMD [2]. Unprecedented visual gains were obtained with monthly intravitreal ranibizumab in the pivotal randomized clinical trials [3,4] and led to a paradigm shift in the treatment of neovascular AMD. However, monthly injections for the management of neovascular AMD patients seen in clinical practice may be difficult to maintain for extended periods [5]. The favorable results predicted for *pro re nata* (PRN) regimens by the results of clinical trials may not apply to what actually occurs in clinical practice over long-term treatment due to the difficulty of maintaining monthly visits [6]. An alternative individualized treatment strategy known as a Treat-and-Extend Regimen (TER) can reduce both the number of injections as compared to fixed monthly therapy and office visits and ancillary testing as compared to both fixed monthly and PRN regimen [7,8]. The TER has now become the regimen used most frequently in the United States.

While the initial major clinical trials for anti-vascular endothelial growth factor (VEGF) therapy in neovascular AMD did not include a TER [3,4,9,10], more recent studies have demonstrated the TER’s ability to yield favorable visual acuity and safety results in patients treated for up to 1 [11,12], 2 [13,14,15] and 3 years [7,16,17]. Moreover, a review of published studies comparing TER and PRN approaches in patients with neovascular AMD [18,19] performed for a consensus manuscript on the TER found that TER was associated with greater (and possibly earlier) visual improvement compared to PRN regimens [8]. The value of the TER still needs validation in larger patient cohorts with long-term follow-up [8]. Therefore, the first objective of this study was to determine if a TER can maintain long-term visual benefits in newly diagnosed treatment-naive neovascular AMD patients treated by a single physician (K. Bailey Freund) over a six-year time period.

The second objective of this study was to correlate baseline demographic and clinical factors with visual changes and long-term visual outcomes. In order to refine the exploration for correlations between baseline lesion composition with visual outcomes, neovascular lesion subtypes were graded using two different classification schemes: the original fluorescein angiography (FA) classification system defined two decades ago by the Macular Photocoagulation Study (MPS) [20] which continues to be used in all major clinical trials and the anatomic classification based on multimodal imaging combining both fluorescein angiography (FA) and optical coherence tomography (OCT). The anatomic classification which is based upon Grossniklaus and Gass’ original observations from histopathology [21] has been described by Freund and associates [22]. The clinical relevance of the anatomic classification in neovascular AMD has recently been demonstrated in two reports analyzing the same cohort of patients studied in this manuscript [23,24]. Herein, we explore the clinical relevance of using the anatomic classification in neovascular AMD by comparing the correlations between long-term visual outcome and neovascular subtypes graded with both classification schemes.

## 2. Methods

This retrospective cohort study design (#20070317, 3 February 2015) was approved by the Western Institutional Review Board (Olympia, WA, USA). It complied with the Health Insurance Portability and Accountability Act of 1996 and followed the tenets of the Declaration of Helsinki.

### 2.1. Data Collection

We retrospectively reviewed the charts and imaging data for a consecutive series of 374 patients who initiated intravitreal anti-VEGF therapy for newly diagnosed, treatment naïve neovascular AMD in one or both eyes between 1 January 2006 and 1 December 2011 and had at least 47 weeks of follow-up by a single physician (K. Bailey Freund) at two offices of the private practice of Vitreous Retina Macula Consultants of New York, New York, NY, USA.

Inclusion criteria were similar to the MARINA [3] and the ANCHOR [4] study groups. All participants were older than 50 years with newly diagnosed treatment-naïve neovascular AMD, as evidenced by clinical examination and FA. Baseline visual acuity (VA) (best recorded distance VA with spectacles, manifest refraction, and/or pinholes) was 20/20 to 20/800 on a Snellen chart (differed from ANCHOR/MARINA which included 20/40 to 20/320 on the Early Treatment Diabetic Retinopathy Study charts). Additionally, eyes in this study must have had OCT imaging (time-domain or spectral-domain) performed at the time of initial diagnosis.

Exclusion criteria were any of the following: previous treatments for choroidal neovascularization (CNV) in the study eye including photodynamic therapy (PDT), intravitreal steroids, intravitreal pegaptanib, or thermal laser; and eyes with CNV lesions with subfoveal fibrosis, central geographic atrophy (GA) at baseline, retinal pigment epithelial tears, or composed of more than 50% hemorrhage. Eyes with CNV secondary to other maculopathies including degenerative myopia, angioid streaks, presumed ocular histoplasmosis syndrome, or inflammatory maculopathies were excluded.

Demographic information including age, gender, race, family history of AMD, smoking status (current, former, never), history of hypertension and diabetes, history of statin, aspirin, clopidogrel, and/or warfarin use, and history of glaucoma were collected for each patient. The number of anti-VEGF injections an eye received, along with best-corrected visual acuity (Snellen) and intraocular pressure at each time point, were recorded from the chart review. The time points evaluated were baseline, 3, and 6 months, followed by every 6 months until last visit available to a maximum follow-up of 72 months. Visual acuity was converted from Snellen chart to logarithm of the minimum angle of resolution (logMAR) for statistical analysis. The type of anti-VEGF agent used for each patient was recorded. Surgical events occurring during the period of the analysis were recorded. The retention rate refers to the proportion of patients who were not lost to follow-up or switched to another treatment strategy before final data collection. Whenever possible, the reason for patients dropping out of treatment or the TER was ascertained from the medical record and/or by contacting the patient directly.

### 2.2. Injection Technique

All injections were performed under topical anesthesia. Prior to injection, the eye was treated with topical proparacaine hydrochloride (0.5%) and topical 5% povidone-iodine solution. Injections were performed 3.5–4.0 mm posterior to the limbus with either a 30-gauge or 32-gauge needle (ranibizumab (0.5 mg/0.05 mL) and aflibercept (2.0 mg/0.05 mL)) or a 31-gauge needle (bevacizumab (1.25 mg/0.05 mL)). No paracenteses or intraocular pressure measurements were performed immediately after injection. All eyes received 3 monthly (4–6 weeks apart) loading doses and were subsequently managed with a TER as described previously, in which maintenance therapy was given while attempting to increase the time interval between treatments [7]. The maximum interval between maintenance injections was typically 9 weeks.

### 2.3. Image Grading

FA images were obtained using a Topcon TRC 50IX fundus camera (Topcon Imagenet, Tokyo, Japan). OCT imaging of all patients was performed with time-domain OCT (Stratus, Carl Zeiss Meditec Inc., Dublin, CA, USA) or spectral-domain OCT (Spectralis, Heidelberg Engineering, Heidelberg, Germany; or 3-D OCT-2000, Topcon, Tokyo, Japan). The OCT data were used for grading of lesion subtype in the anatomic classification of lesion composition [22]. Standard methods of image acquisition were employed for all imaging modalities.

The classification of neovascular lesions was made independently by two experienced retina specialists (Sarah Mrejen and Roberto Gallego**-**Pinazo) who evaluated the presenting color photographs, FA, and OCT. First, all the color photographs and FA corresponding to the baseline diagnostic visit were analyzed. Neovascular lesions were subtyped according to the MPS criteria [20] and the Digital Angiographic Reading Center Reader’s Manual as occult or classic CNV, and retinal angiomatous proliferation (RAP) lesions were identified by criteria defined by Yannuzzi *et al*. [25] and the Digital Angiographic Reading Center Reader’s Manual. Secondly, OCT images corresponding to the same diagnostic visit were reviewed, and each case was classified according to the guidelines provided by Freund *et al.* [22]. The anatomic classification, which utilizes OCT in combination with FA, categorizes lesions as Type 1 (sub-RPE), Type 2 (subretinal), Type 3 (intraretinal), or Type 4 (mixed lesions) neovascularization (NV). Eyes with polypoidal choroidal vasculopathy (PCV) were considered to be a form of Type 1 NV. Cases with multiple lesion types were identified as mixed NV and each component was also recorded. The specific criteria used in the anatomic classification have been previously described [22,23]. Finally, in cases with disagreement between FA and OCT findings, FA images were re-analyzed paying special attention to the early frames in order to better recognize subtle angiographic findings, in particular those of Type 3/RAP lesions that might have not been recognized during the first analysis of the images. A third supervising grader (K. Bailey Freund) evaluated the lesion type in the presence of significant discrepancies.

Readers also graded the lesion location and overall size. FA was used to measure the greatest linear dimension (mm) and the total area of CNV lesion (mm²). Measurements were performed only on fundus camera images. The total area of CNV lesion was defined as the area of CNV leakage plus any contiguous areas of thick hemorrhage, blocked fluorescence, or serous PED that could be obscuring the boundaries of the CNV. The lesion location was defined as subfoveal, juxtafoveal, or extrafoveal as determined according to the MPS terminology [20]. Specifically, foveal location was defined as the most posterior border of the lesion, including blood or blocked fluorescence involving the geometrical center of the fovea as observed on FA.

### 2.4. Statistical Analysis

Baseline predictors were measured on a categorical scale (anatomic classification, FA classification, lesion localization) or on a continuous scale (logMAR VA, lesion area, and number of injections). Since the duration of follow-up was heterogeneous mainly due to inclusion of patients at different time points between 2006 and 2011, the analysis was done at each time point and not final follow-up.

We analyzed predictors for 4 VA outcomes, including VA score, change in VA score from baseline, a gain of ≥3 lines (*i.e*., 15 letters) from baseline, and a loss of ≤1 line (*i.e*., 5 letters) from baseline. Bivariate relationships between the 4 VA outcomes and the various baseline parameters were evaluated without adjustment for other covariates using the two-sample *t*-test, Wilcoxon rank sum test, or Spearman’s correlation for continuous variables, and the chi-square test, Fisher’s exact test, or one way analysis of variance (ANOVA) for categorical variables. Bivariate analysis was repeated at time points of interest (6 months, 1, 2, 3, 4, 5 and 6 years) in which there was enough statistical power for assessment. To compare quantitative variables among the different groups for multiple comparisons, one way ANOVA was used with Games-Howell for unequal variances corrections and with Bonferroni for equal variances corrections. Bilaterality was evaluated as a parameter in the bivariate analysis, but this does not completely solve the problem of bilaterality since two eyes from the same patient cannot be considered independent.

Specific clinical characteristics were further assessed for a potential independent effect on the VA outcomes of interest. The number of subjects in the different outcome groups limited the number of variables that could be explored in the multivariate model. Based on the bivariate analyses and *a priori* considerations, certain variables were not included for further evaluation. Variables that resulted in a *p*-value <0.2 from the bivariate analyses at various time points were further evaluated using a generalized linear model for VA and change in VA from baseline. An assessment of collinearity between predictors was performed prior to the specification of the final multivariate model. All *p*-values were two-sided with statistical significance at the 0.05 alpha level. Beta (β) and standard error (SE) were constructed to assess the precision of the obtained estimates. All data were analyzed in SPSS Version 22 (SPSS Inc., Chicago, IL, USA).

## 3. Results

A total of 374 patients initiated anti-VEGF therapy for newly diagnosed treatment-naïve neovascular AMD between 1 January, 2006 and 1 December 2011. Among these 374 patients, 185 patients (210 eyes) met the eligibility criteria.

### 3.1. Baseline Demographic Characteristics and Correlation with Visual Outcome

Among the 185 patients, the mean age ±SD at first injection was 81.1 ± 8.0 years; 13.5% of patients (25/185) had bilateral study-eligible disease; 67.6% (125/185) were women; 95.1% (176/185) were white, followed by Hispanic 2.2% (4/185), Asian 2.2% (4/185), and Black 0.5% (1/185). Among the 185 patients, 70.8% (131/185) had no history of smoking, 23.8% (44/185) were former smokers and 5.4% (10/185) current smokers. There was a family history of AMD in 18.9% of cases (35/185), a history of hypertension in 61.1% (113/185), diabetes in 10.8% (20/185); 37.3% (69/185) were using statin medication, 35.7% (66/185) aspirin, 5.9% (11/185) clopidogrel, and 8.6% (16/185) warfarin. There was a history of glaucoma in 8.6% (16/185) of patients.

The correlations between baseline demographic characteristics and visual outcomes were evaluated at all time points (details not shown). The bivariate analysis revealed that an older age at first injection was correlated with poorer visual acuity at 1, 2, 3 and 4 years. Hypertension was correlated with poorer visual outcome at baseline, 1, 2 and 5 years. Aspirin intake was correlated with poorer visual acuity at 1 year and 6 years. Clopidogrel intake was correlated with poorer visual acuity at 6 years. There was no significant correlation with any of the demographic variables and visual change at any time point (data not shown). Bilaterality was evaluated as a parameter in the bivariate analysis and was not significantly associated with VA or change in VA at any time point evaluated.

### 3.2. Neovascular Lesions and Number of Injections

The distribution of neovascular lesion subtypes according to anatomic classification was: 38.6% of Type 1 NV, 8.6% of Type 2 NV, 34.3% of Type 3 NV and 18.6% of Type 4 (mixed lesions) NV. Patients received a mean of 28.5 ± 14.3 injections over a mean follow-up period of 3.5 ± 1.67 years. The mean number of injections per year was 8.3 ± 1.6 (range 4 to 13) and the mean interval between injections was 6.6 ± 1.5 weeks. The mean number of injections per year was 8.8, 7.7, 8.1, and 7.8 for Type 1, 2, 3 and 4 NV respectively. The mean number of injections according to lesion composition is detailed in Table 2. The neovascular lesion localization was foveal in 63% (133/210), juxtafoveal in 18% (37/210), and extrafoveal in 19% (40/210) of eyes. The mean overall lesion area was 6.6 ± 5.9 mm² and the mean greatest lesion diameter 3.2 ± 1.5 mm. The majority, 59% (124/210), received ranibizumab only treatment, and the remainder received either a combination of ranibizumab and aflibercept 15.7% (33/210), ranibizumab and bevacizumab 14.3% (30/210), all three agents 5.7% (12/210), bevacizumab alone 4.3% (9/210) or aflibercept alone 1% (2/210). Thirty out of 210 eyes (14.3%) had cataract extraction over the 6 year time period.

**Table 1:** Bivariate associations of baseline clinical characteristics and visual parameters at 3 years.

Baseline Characteristics *N* = 128 Eyes	VA at 3 Years (logMAR)		VA Change at 3 Years (logMAR)		≥3 Line Gainat 3 Years		≥1 Line Lossat 3 Years	
	Adjusted Mean (SE)	*p* Value	Adjusted Mean (SE)	*p* Value	Number (%)	*p* Value	Number (%)	*p* Value
**Baseline VA**	-	**<0.001**	-	**<0.001**	-	**<0.001**	-	0.11
**6-month VA**	-	**<0.001**	-	0.068	-	0.979	-	0.083
**Total**	0.544 (0.037)		−0.106 (0.033)		38 (28%)		28 (22%)	
**Anatomic classification**		**0.006**		**0.001**		**0.041**		0.478
**1**	0.390 (0.050)		−0.113 (0.048)		18 (47%)		9 (32%)	
**2**	0.695 (0.129)		−0.215 (0.123)		6 (16%)		3 (11%)	
**3**	0.534 (0.055)		−0.060 (0.051)		9 (24%)		9 (32%)	
**4**	0.761 (0.096)		−0.109 (0.087)		5 (13%)		7 (25%)	
**Fluorescein classification**	0.544 (0.037)	0.214	−0.106 (0.033)	0.194		0.521		0.613
**1**	0.463 (0.051)		−0.076 (0.043)		18 (47%)		13 (46%)	
**2**	0.651 (0.109)		−0.269 (0.100)		8 (21%)		3 (11%)	
**3**	0.606 (0.066)		−0.068 (0.064)		9 (24%)		8 (29%)	
**4**	0.593 (0.122)		−0.094 (0.098)		3 (8%)		4 (14%)	
**Lesion location**		0.841		**0.023**		0.09		**0.01**
**foveal**	0.552 (0.045)		−0.166 (0.041)		31 (82%)		14 (50%)	
**juxta**	0.559 (0.082)		0.001 (0.065)		3 (8%)		6 (21%)	
**extra**	0.490 (0.104)		0.048 (0.066)		4 (11%)		8 (29%)	
**Lesion area**	-	0.301	-	0.039	-	0.138	-	0.298
**Number of injections**	-	**<0.001**	-	**0.022**	-	0.259	-	0.104

VA: visual acuity; logMAR: logarithm of the minimum angle of resolution; SE: standard error.

**Table 2:** Mean number of injections broken down yearly according to neovascular lesion type.

Neovascular Lesion Type (FA + OCT)	Mean Number of Injections per Year					
	1st Year	2nd Year	3rd Year	4th Year	5th Year	6th Year
Type 1	9.4	8.2	8.6	8.6	8.5	9.1
Type 2	8.3	6	7.8	7.8	8.1	8.2
Type 3	8.7	8	7.5	8.1	7.8	8.4
Type 4 (mixed)	8.8	7.3	7.4	6.8	7.9	7

FA: fluorescein angiography; OCT: optical coherence tomography.

### 3.3. Retention Rate

The retention rate for the entire cohort was 62.9% over the 6 year time period (132/210 eyes). The reasons for the 78 eyes falling out of the TER were as follows: death (20), transferred care elsewhere due to relocation (20), switched to PRN strategy at the treating physician’s discretion due to risk of progressive geographic atrophy (14) and missed or delayed appointment due to unrelated systemic illness (7). Only 17 of 210 eyes (8.1%) were lost to follow-up for unknown reason. The retention rate was 100% (210/210) at 1 year, 83% (174/210) at 2 years, 61% (128/210) at 3 years, 42% (88/210) at 4 years, 26% (54/210) at 5 years and 13% (27/210) at 6 years.

### 3.4. Visual Results for the Entire Cohort

**Figure 1:**
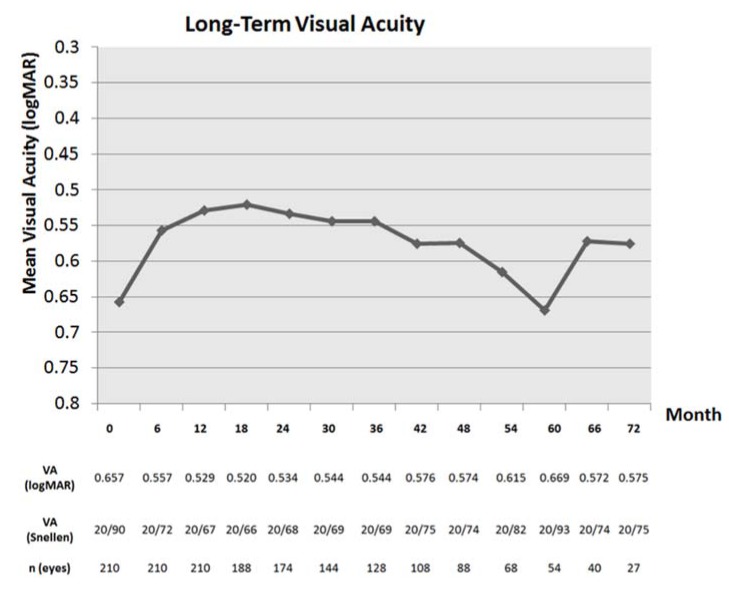
Mean visual acuity over time. The visual acuity is noted in logMAR and Snellen chart, as well as the number of eyes at each time point.

**Figure 2:**
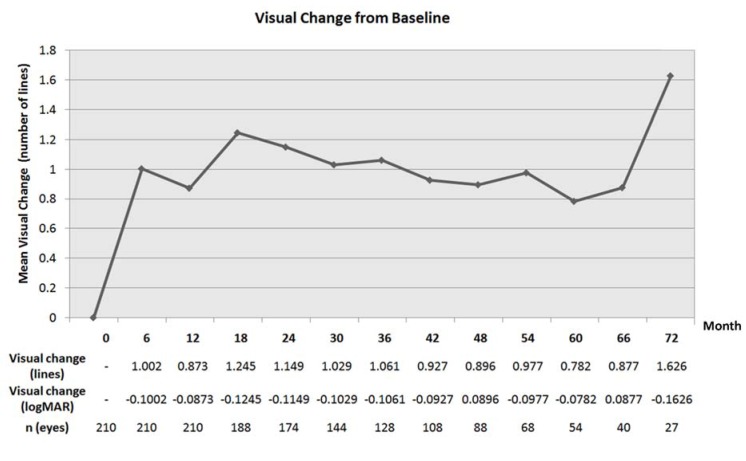
Mean visual change from baseline over time. The mean change in visual acuity from baseline is noted in logMAR and equivalent of lines.

The results of mean visual acuity for the entire cohort are detailed in Figure 1. Visual benefits obtained at 6 months were largely maintained long-term at 3, 4, and 6 years, although, at 5 years, there was a decline in mean VA approaching baseline VA, which appears inconsistent with the rest of the curve. For the entire cohort, the mean visual changes from baseline are detailed in Figure 2. Maximal visual benefits from baseline were seen at 18 months (−0.1245 logMAR, equivalent of 1.245 lines). Despite a slight decrease long term, these visual benefits were maintained with a visual gain of −0.1061, −0.0896, and −0.0782 logMAR at 3, 4, and 5 years respectively. At 6 years, one isolated point shows a mean visual gain of −0.1626 logMAR, not consistent with the rest of the curve (Figure 2). This may be due to a relatively smaller number of patients with very long follow-up, or a biased retention of patients with better visual outcome.

For the entire cohort, the significant clinical predictors of visual acuity at all time points in bivariate analysis were baseline VA, 6 month VA, number of injections, and anatomic classification (Table 1 and Table 3). Baseline and 6 month VA were positively correlated with VA at all time points (6, 12, 24, 36, 48, 60, and 72 months). The fluorescein classification was not correlated with visual acuity at any time point. Using the anatomic classification, eyes with Type 1 NV had significantly better visual acuity than other neovascular subtypes at all time points. The predictors of visual change at all time points were baseline VA and number of injections in bivariate analysis (Table 1 and Table 3). Baseline VA was negatively correlated with visual acuity change at all time points (6, 12, 24, 36, 48, 60, and 72 months).

Patients with worse baseline VA were more likely to improve over time. Overall lesion area was significantly negatively correlated with visual change at 1 year (*p =* 0.012) and 4 years (*p =* 0.043, Table 3). Smaller lesions were more likely to improve in VA. The anatomic classification was correlated with visual change at 1 year (*p =* 0.058, Table 1) and 3 years (*p =* 0.001). The fluorescein classification was correlated with visual change at 1 year (*p =* 0.006, Table 1). Eyes with Type 2 NV had greater visual improvement compared to other neovascular subtypes at 1, 2, and 3 years. At 4 years, eyes with Type 1 NV had greater visual improvement compared to other subtypes. We used independent sample *t*-tests of visual acuity at each time point *versus* presence or absence of a cataract operation before that time point during the study period, and there was no significant association between cataract extraction and visual acuity at any time point.

*Post hoc* adjustments for multiple comparisons revealed a statistically significant difference in anatomic classification between Type 1 and Type 4 (mixed lesions) with visual acuity at 1 year (*p* < 0.001), 2 years (*p* = 0.002), 3 years (*p* < 0.001), and 4 years (*p* = 0.003) (Table 1 and Table 3). No significant differences were noted among the other pairwise comparisons of anatomic classifications (1 *vs*. 2; 1 *vs*. 3; 2 *vs*. 3; 3 *vs*. 4). *Post hoc* adjustments for multiple comparisons revealed a statistically significant difference in fluorescein classification when comparing changes in VA at 1 year from baseline between groups 1 *vs*. 2 (*p* = 0.023); and 2 *vs*. 3 (*p* = 0.004) (Table 1). No significant differences were noted among the other pairwise comparisons of fluorescein classification at other time points.

### 3.5. Visual Results by Neovascular Phenotype

**Figure 3:**
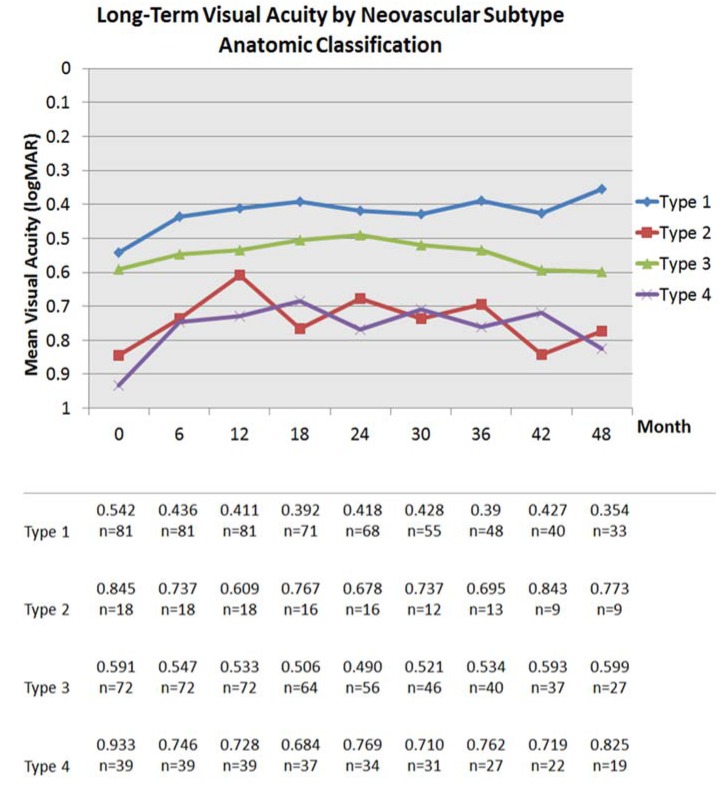
Mean visual acuity in logMAR by neovascular subtype using the anatomic classification. The number of eyes for each neovascular subtype is reported at each time point.

**Figure 4:**
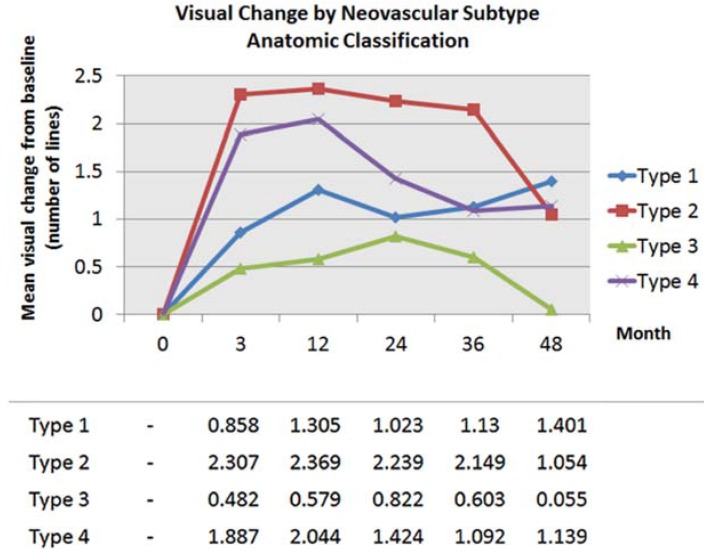
Mean visual change from baseline in line equivalent by neovascular subtype using the anatomic classification.

The visual results over time by neovascular lesion type are detailed in Figure 3 and Table 1 and Table 3. Eyes with Type 1 NV started with better baseline VA, followed by Types 3, 2, and 4 (mixed) NV and this sequence remained true at 1, 2, 3 and 4 years (Figure 3). The visual results by neovascular lesion type at 5 and 6 years are not detailed due to smaller number of patients in each subgroup. The fluctuations of the curve representing visual acuity of patients with Type 2 NV on Figure 3 may be due to smaller number of patients with that neovascular lesion subtype. Figure 4 shows the mean visual change by neovascular lesion type using the anatomic classification. Visual change increased in all subtypes between baseline and 1 year and then decreased slightly in all subtypes except for eyes with Type 1 NV that continued to show visual improvement until 4 years (Figure 4).

**Table 3:** Bivariate associations of baseline clinical variables and visual parameters at 4 years.

Baseline Characteristics *N* = 88 Eyes	VA at 4 Years (logMAR)		VA Change at 4 Years (logMAR)		≥3 Line Gain at 4 Years		≥1 Line Loss at 4 Years	
	Adjusted Mean (SE)	*p* Value	Adjusted Mean (SE)	*p* Value	Number (%)	*p* Value	Number (%)	*p* Value
**Baseline VA**	-	**<0.001**	-	**0.002**	-	**0.001**	-	0.384
**6 month VA**	-	**<0.001**	-	0.092	-	0.661	-	0.169
**Total**	0.574 (0.051)		−0.090 (0.044)		26 (30%)		20 (23%)	
**Anatomic classification**		**0.006**		0.652		0.498		0.314
**1**	0.354 (0.054)		−0.140 (0.054)		11 (42%)		5 (25%)	
**2**	0.773 (0.185)		−0.105 (0.138)		3 (12%)		2 (10%)	
**3**	0.599 (0.074)		−0.006 (0.073)		7 (27%)		9 (45%)	
**4**	0.826 (0.148)		−0.114 (0.137)		5 (19%)		4 (20%)	
**Fluorescein classification**	0.573 (0.051)	0.135	−0.090 (0.044)	0.839		**0.002**		0.831
**1**	0.442 (0.061)		−0.094 (0.048)		10 (39%)		9 (45%)	
**2**	0.713 (0.176)		−0.194 (0.166)		6 (23%)		3 (15%)	
**3**	0.680 (0.089)		−0.022 (0.096)		9 (35%)		7 (35%)	
**4**	0.650 (0.178)		−0.085 (0.107)		1 (4%)		1 (5%)	
**Lesion location**		0.616		0.25		0.073		0.511
**foveal**	0.581 (0.061)		−0.136 (0.056)		23 (89%)		13 (65%)	
**juxta**	0.636 (0.133)		0.022 (0.072)		1 (4%)		4 (20%)	
**extra**	0.449 (0.139)		0.036 (0.081)		2 (8%)		3 (15%)	
**Lesion area**	-	0.592	-	**0.043**	-	**0.049**	-	0.085
**Number of injections**	-	**0.007**	-	0.175	-	0.57	-	0.242

VA: visual acuity; logMAR: logarithm of the minimum angle of resolution; SE: standard error.

### 3.6. Multivariate Analysis

The results of multivariate analysis are detailed in Table 4 for the entire cohort, and for the subgroup of patients with at least 4 years of follow-up (88 eyes) in Table 5. The anatomic classification was included in the multivariate analysis and not the fluorescein classification because the anatomic classification was more consistently and strongly correlated with visual acuity and visual changes over time in the bivariate analysis. For the entire cohort (Table 4), the number of injections and anatomic classification were consistently and independently correlated with visual acuity at all time points. The number of injections was the strongest independent predictor of visual acuity at all time points. Eyes with Type 1 NV had better visual outcomes compared to the other neovascular phenotypes. The number of injections was also an independent predictor of visual improvement at 6 months (*p =* 0.054) and 1 year (*p =* 0.033). Overall lesion area was an independent predictor of visual acuity at 1 (*p =* 0.019), 2 (*p =* 0.003), and 3 years (*p =* 0.049). Smaller lesions were more likely to have better visual acuity. Lesion localization was an independent predictor of visual change at 1 (*p =* 0.044), 2 (*p =* 0.045), and 3 years (*p =* 0.007). Subfoveal neovascular lesions were more likely to show visual improvement compared to extrafoveal lesions. For the subgroup of patients with at least 4 years of follow-up, the results were similar to the entire cohort (Table 5) The anatomic classification and number of injections were again independent predictors of visual acuity at all time points in the multivariate analysis. However, overall lesion area and lesion localization were not significant independent predictors of visual acuity or visual acuity change.

**Table 4:** Multivariate analysis of visual acuity and visual acuity change using baseline clinical parameters over time.

Time Point	Visual Parameter		Anatomic Classification	Number of Injections	CNV Localization	Overall Lesion Area	Age at First Injection
**6 months**	**VA**	B	0.06	−0.05	−0.04	0.01	0.01
		SE	0.03	0.02	0.04	0.01	0.00
		*p*	**0.02**	**0.00**	0.26	0.09	**0.05**
	**VA Change**	B	−0.01	−0.03	0.05	−0.01	0.00
		SE	0.02	0.01	0.03	0.00	0.00
		*p*	0.55	0.05	0.12	0.15	0.55
**12 months**	**VA**	B	0.07	−0.06	−0.03	0.01	0.01
		SE	0.03	0.02	0.04	0.01	0.00
		*p*	**0.01**	**0.00**	0.50	**0.02**	0.06
	**VA Change**	B	−0.01	−0.03	0.06	0.00	0.00
		SE	0.02	0.02	0.03	0.00	0.00
		*p*	0.79	**0.03**	**0.04**	0.67	0.58
**24 months**	**VA**	B	0.06	−0.07	−0.02	0.02	0.01
		SE	0.03	0.02	0.05	0.01	0.00
		*p*	**0.04**	**0.00**	0.71	**0.00**	0.07
	**VA Change**	B	−0.01	−0.03	0.08	0.01	0.00
		SE	0.02	0.02	0.04	0.01	0.00
		*p*	0.82	0.06	**0.05**	0.34	0.26
**36 months**	**VA**	B	0.09	−0.09	0.02	0.01	-
		SE	0.03	0.02	0.05	0.01	-
		*p*	**0.00**	**<0.001**	0.65	**0.05**	-
	**VA Change**	B	0.02	−0.03	0.13	0.00	-
		SE	0.03	0.02	0.05	0.01	-
		*p*	0.47	0.11	**0.01**	0.90	-
**48 months**	**VA**	B	0.11	−0.11	0.01	0.00	-
		SE	0.04	0.03	0.07	0.01	-
		*p*	**0.01**	**0.00**	0.87	0.89	-
	**VA Change**	B	0.02	−0.05	0.10	−0.01	-
		SE	0.04	0.03	0.07	0.01	-
		*p*	0.66	0.09	0.15	0.13	-

CNV: choroidal neovascularization; VA: visual acuity; B: beta coefficient; SE: standard error; *p*: *p*-value.

**Table 5:** Multivariate analysis of visual acuity and visual acuity change using baseline clinical parameters over time using patients with at least 4 years of follow up (*N* = 88).

*p Values*						
Time Point	Visual Parameter	Anatomic Classification	Number of Injections	CNV Localization	Overall Lesion Area	Age at First Injection
6 months	VA	**0.043**	**0.016**	0.812	0.564	0.496
	VA Change	0.674	0.445	0.056	0.291	0.492
12 months	VA	**0.048**	**0.050**	0.859	0.627	0.294
	VA Change	0.591	0.856	0.086	0.277	0.286
24 months	VA	**0.024**	**0.026**	0.976	0.609	0.193
	VA Change	0.367	0.455	0.110	0.294	0.202
36 months	VA	**0.021**	**0.010**	0.504	0.769	-
	VA Change	0.874	0.271	**0.020**	0.211	-
48 months	VA	**0.011**	**0.002**	0.871	0.887	-
	VA Change	0.661	0.090	0.151	0.127	-

CNV: choroidal neovascularization; VA: visual acuity.

### 3.7. Intraocular Pressure and Safety

The mean intraocular pressure (IOP) at baseline was 14.4 ± 2.8 mm Hg. There were no IOP elevations greater than 31 mm Hg recorded at any of the study time points and no eyes with sustained IOP elevations above 25 mm Hg. There were no injection-related complications such as endophthalmitis and retinal detachment. No serious systemic adverse events were recorded in the charts.

## 4. Discussion

We present one of the largest cohorts of neovascular AMD patients managed with a TER of intravitreal anti-VEGF therapy. This consecutive series was treated by a single physician (K. Bailey Freund) over a 6 year period during which nearly all eyes with treatment-naive neovascular AMD were managed with a TER. The mean follow-up of 42 months is longer than that of previous reports on the use of a TER for this diagnosis. In addition, the retention rate of 62.9% is considerably higher than that of similar long-term follow-up studies [26,27].

In order to compare our results to those of eyes treated in the large randomized trials of anti-VEGF therapy for neovascular AMD, we used inclusion criteria similar to those used in ANCHOR, MARINA, and other large clinical trials exploring intravitreal anti-VEGF therapy for neovascular AMD [3,4,9,10]. However, unlike some of these trials, we chose to include eyes with all neovascular lesion sub-types including type 3 NV/RAP and PCV (considered as variant of type 1 NV/occult lesions). While PCV lesions were uncommon in our cohort (3.3% of eyes), type 3 NV/RAP accounted for 34% of the treatment-naive cases meeting our inclusion criteria. As type 3 NV/RAP eyes had somewhat worse visual outcomes than the mean VA for all lesion types, their inclusion resulted in worse mean visual acuities at each time point. Nevertheless, the mean visual change of −0.115 logMAR (equivalent of +1.15 lines) at 2 years in our cohort was similar to that for eyes in the MARINA and ANCHOR trials at 2 years eyes, which improved by 7.2 letters and 10.7 letters, respectively. Other VA efficacy endpoints were comparable as well, with 30% achieving 3 line gains (38% for eyes with Type 1 NV) at 2 years, compared to 33.8% and 41% in the MARINA and ANCHOR trials, respectively [3,4].

The visual benefits achieved at 1 and 2 years were largely maintained with continued therapy after a mean follow-up of the entire cohort of 3.5 years. In the subgroup of patients followed for 4 (*n =* 88) and 6 years (*n =* 27), the visual benefits were maintained as well, but these results will need to be confirmed in larger series of patients managed with a TER. As the follow-up extended toward 5 to 6 years, there were fewer eyes available for analysis likely leading to fluctuation in overall visual acuity and in the standard deviations at each time point (Figure 1), but the overall trend demonstrated an initial improvement similar to the other major treatment studies with general trend toward stabilization with minimal loss. Whereas patients in the MARINA and ANCHOR trials were examined and treated every 4 weeks, our patients were examined less often and received injections a mean of every 6.3 weeks (8.3 injections per year).

There is limited data available on long-term follow-up beyond 2 years in patients with neovascular AMD treated with anti-VEGF, and our results compare favorably to the available published literature. Most published long-term studies have reported unchanged visual acuity compared with baseline acuity after 3, 4 [27,28], and 7 years [26], and consistently found a gradual visual loss over time from the initial benefits obtained at 2 years [26,27,28]. The long-term data from HORIZON [27], SECURE [29], and SEVEN-UP [26] all suggested that the incremental decline of vision over time may be due to underlying disease progression or decrease in injection frequency over time [26,27]. Each of these open-label extension studies also had significant loss of patients by 5–6 years that may have also influenced these visual outcomes. As opposed to the HORIZON study in which the patients were seen every 3 months and treated at the discretion of the investigator [27], in the open-label extension of the VIEW 1 study, aflibercept injections were repeated at least every 12 weeks which appears to have helped to maintain visual benefits of +7.0 letters at 192 weeks in 323 patients, suggesting the possibility of maintaining long-term visual benefits with continuous anti-VEGF therapy [30]. Recently, Rayess and colleagues [17] followed a cohort of 212 eyes from 196 patients over 3 years with a TER; and although they also lost a significant proportion of eyes and only analyzed 27.8% (59/212) eyes at 3 years, they confirmed the possible long-term visual and anatomical benefits of TER. Our extended follow-up data from a single practitioner utilizing a uniform TER help confirm the potential to maintain long-term visual gains by maintaining continuous anti-VEGF therapy and adjusting the interval to treatment response through the TER approach. It is possible that this strategy results in better long-term visual outcomes compared to PRN regimens by reducing the risk of complications associated with recurrent neovascular activity such as catastrophic macular hemorrhages. In addition, continuous therapy might minimize the potential for continued growth and maturation of neovascular membranes that might lead to subsequent treatment resistance [31], but parameters such as these were not part of the outcome measures in this analysis.

The number of intravitreal anti-VEGF injections has been consistently positively correlated with visual outcome in numerous studies including the current analysis [9]. Even though the association between number of injections and visual outcome has not always been analyzed in long-term studies [27], the SEVEN-UP study found that, after seven years of follow-up, the subgroup of patients who received more anti-VEGF injections had significantly better visual gains [26]. However, the number of anti-VEGF injections has recently been demonstrated to be significantly correlated with the development of GA in neovascular AMD [9,32]. As a greater number of injections correlates with both better visual outcomes and a higher rate of GA, an individualized retreatment strategy may be an attractive alternative to potentially strike a balance between too many and too few injections.

In our analysis of the association between baseline lesion composition and long-term visual outcomes, eyes with Type 1 NV appeared to do the best and were the only group that maintained long-term visual gains. Eyes with other lesion subtypes incrementally lost vision after an initial phase of visual improvement (Figure 3 and Figure 4). Xu *et al.* [24] evaluated the risk of developing GA in this same cohort of patients and found that the eyes with Type 1 NV were 6.7 times less likely to develop GA compared to the other neovascular subtypes. These results suggest that the assessment of the neovascular lesion subtype may be helpful for an individualized therapeutic strategy. For example, several studies have found an association between Type 3 NV/RAP lesions and GA development in eyes receiving intravitreal anti-VEGF therapy [32,33] and in the fellow untreated non-neovascular eye [34]. The sub-analysis of the Comparison of Age-related Macular Degeneration Treatments Trials (CATT) study demonstrated that the presence of a RAP lesion was an independent predictor of developing GA at 2 years [32]. In our analysis, eyes with Type 3 NV and eyes with Type 4 NV (mixed lesions) that included a Type 3 NV component may have lost the initial visual benefits obtained at 2 years due to the development of GA or due to central progression of pre-existing GA (eyes with central GA at baseline were excluded from this analysis). The sub-analysis of the CATT study assessing the risk of scarring found that eyes with classic CNV were more likely to develop scarring at 2 years [35]. In this analysis, eyes with Type 2 NV and eyes with Type 4 (mixed lesions) NV including a Type 2 component may have also lost the initial visual benefits due to scarring. In a histopathological study of AMD eyes with disciform scars, Green reported that 37% had areolar atrophy associated with the scar, indicating that GA and scarring are not mutually exclusive complications in neovascular AMD [36].

In our study, eyes with Type 1 NV received more frequent anti-VEGF injections than other NV types, presumably due to more refractory exudative signs. Despite some refractory subretinal fluid (SRF), eyes with Type 1 NV had the best visual outcomes and developed less GA than eyes with other lesions subtypes. A possible explanation for these results may be that sub-RPE NV is a more mature form of NV with a slower growth potential. In addition, Grossniklaus and Green have hypothesized that sub-RPE NV is a compensatory form of neovascularization that could provide nutrients and oxygen to the RPE and outer retina [37]. There may be nutrients in the chronic SRF that help maintain photoreceptor integrity [38]. Perhaps, for these reasons, eyes with Type 1 NV are more likely to have long-term benefit from long-term maintenance anti-VEGF therapy with a TER approach than eyes with other lesions. Conversely, eyes with Type 2, 3, and 4 (mixed) NV may be more prone to develop GA and scarring and be less likely to maintain long-term visual benefits with long-term anti-VEGF therapy on a TER approach.

In order to individualize therapy based on initial lesion composition, there is a need for an accurate and reproducible method to assess lesion composition. In this study, the anatomic classification correlated more closely than the fluorescein classification with visual outcomes at all time points evaluated from baseline to 4 years (Table 1 and Table 3). The anatomic classification was also an independent predictor of visual acuity at all time points in the multivariate analysis (Table 4 and Table 5). Based on fluorescein alone, Cohen and associates showed that there was only moderate inter-grader agreement [39]. Jung and associates analyzed the data of this cohort using FA alone *versus* combined FA and OCT and concluded that the anatomic classification may be a more accurate and reliable way to assess neovascular phenotype [23]. They also showed that the addition of OCT to FA allowed the two readers to make a more precise identification of Type 3 NV/RAP and mixed lesions [23]. As mentioned above, the clinical relevance of the anatomic classification has also been demonstrated in an analysis of this same cohort finding a stronger correlation between GA progression and neovascular lesion type when using the anatomic classification compared to FA classification [24]. We report a frequency of Type 3 NV/RAP lesions as high as 34% in our cohort. It is noteworthy that this neovascular subtype has rarely been analyzed as a separate category in the major neovascular AMD treatment trials and these eyes may have been included the cohorts of eyes graded as having occult or minimally classic lesions based on FA grading [4,9]. The main report of the CATT [9] did not mention RAP lesions but, in the sub-analysis assessing the risk of GA, the entire cohort has been categorized into predominantly classic, minimally classic, and occult lesions, and the presence of RAP has been noted as a separate finding [32]. In the CATT report, it is not mentioned exactly how RAP lesions were diagnosed, whether it was by OCT, FA, or a combination of both [32].

There has been some concern regarding the higher number of injections in a TER approach and subsequent increased risk of complications. One possibility is an increased risk for sustained IOP elevation with greater number of injections.[40,41] In this report, there were no IOP elevations greater than 31 mmHg at all time points and no sustained IOP elevations above 25 mm Hg. There were no cases of endophthalmitis, retinal detachment, or other serious ocular or systemic adverse events.

We acknowledge that there are inherent limitations of this study including its retrospective nature, the use of different OCT devices to classify the neovascular lesions and use of different anti-VEGF agents. Some limitations were intrinsic to the very long follow-up period up to 6.6 years such as changes in the treatments available during this period and fewer number of patients at each extended follow-up periods of 5 and 6 years. For example, some patients were first evaluated before the availability of spectral domain OCT and had time domain OCT at baseline. In addition, patients treated with aflibercept soon after its commercial approval may have been managed differently due to the impression that this drug may have a more durable effect. The choice of drug may not have been independent of baseline characteristics. It is possible that larger lesion size would fail extension and benefit being switched to aflibercept. Therefore, the choice of drug may have been a confounder for the visual outcome. Moreover, a greater proportion of eyes enrolled later in the enrollment period may have been treated with aflibercept and have shorter follow-up. Another limitation was that a small number of eyes were switched to a PRN regimen (6.7%) due to concerns regarding a theoretical increased risk for the development of or progression of central geographic atrophy. This subgroup of patients included a disproportionate number of eyes with Type 2, 3 and mixed lesions that may have been at a higher risk for a poorer visual outcome therefore potentially biasing toward good visual outcomes. The minimum follow-up was only 1 year, which is relatively short for a long-term analysis, but a separate analysis of the subgroup of patients with at least 4 years of follow-up (*N* = 88 eyes) found similar results (Table 5). A small proportion of patients underwent cataract surgery over the study period, which could affect the visual acuity outcomes, although no statistically significant association was found between visual acuity and cataract operation. Additionally, there were 13.5% bilateral cases in which injections were typically given to both eyes on the same day. In these cases, the eye requiring more frequent injections often dictated the TER interval. Therefore, the treatment interval in these patients may have been biased to a shorter length. Moreover, eyes from the same patient cannot be considered independent and a non-significant result in a bivariate analysis does not completely solve this problem. Therefore including both eyes from some patients remains a weakness of our study. Despite these limitations, it has been possible to draw a number of statistically sound conclusions that we believe are of significant value in guiding real-word clinical practice.

In this retrospective study, a TER for eyes with treatment-naive neovascular AMD produced long-term visual benefits with a mean of 8.3 injections per year. While we acknowledge a potential bias of patients with better visual outcomes being more likely to remain on the prescribed regimen than those doing poorly, our retention rate of 62.9% is considerably higher than similar cohorts, possibly indicating that the TER strategy may actually improve patient compliance by reducing the burden of frequent visits compared to PRN dosing strategies. Moreover, the reasons why patients fell out of the TER were well documented for the majority of eyes and was not due to a poor visual prognosis but to death, relocation or unrelated systemic illness delaying an appointment. There were only 8.1% lost to follow-up for unknown reasons, likely minimally influencing the long-term visual outcomes.

As eyes with Type 1 NV had better visual outcomes than other neovascular subtypes despite requiring more frequent injections,, our results suggest that individualizing anti-VEGF therapy based on initial lesion composition may be of value. The anatomic classification was more strongly correlated with visual outcome than the fluorescein classification and one of the only 2 independent predictors of visual outcome at all time points, the other being a higher number of injections. 

## 5. Conclusions

In conclusion, our results presented here and in other published findings from this same patient cohort [23,24] suggest that an anatomic classification of neovascular lesion composition based on multimodal imaging AMD may be more clinically meaningful than the fluorescein agiography-based classification and may be useful in guiding individualized therapeutic dosing strategies including the TER. A TER provided sustained long-term visual gains with a high patient retention rate. Eyes with Type 1 neovascularization had better visual outcomes than those with other neovascular lesion subtypes.
